# Identifying association model for single-nucleotide polymorphisms of *ORAI1* gene for breast cancer

**DOI:** 10.1186/1475-2867-14-29

**Published:** 2014-03-31

**Authors:** Wei-Chiao Chang, Yong-Yuan Fang, Hsueh-Wei Chang, Li-Yeh Chuang, Yu-Da Lin, Ming-Feng Hou, Cheng-Hong Yang

**Affiliations:** 1 Master Program for Clinical Pharmacogenomics and Pharmacoproteomics, School of Pharmacy, Taipei Medical University, Taipei, Taiwan; 2 Department of Clinical Pharmacy, School of Pharmacy, Taipei Medical University, Taipei, Taiwan; 3 Cancer Center, Kaohsiung Medical University Hospital, Kaohsiung, Taiwan; 4 Labor Safety and Health Office, Kaohsiung Municipal Ta-Tung Hospital, Kaohsiung, Taiwan; 5 Department of Biomedical Science and Environmental Biology, Kaohsiung Medical University, Kaohsiung, Taiwan; 6 Institute of Medical Science and Technology, National Sun Yat-sen University, Kaohsiung, Taiwan; 7 Department of Chemical Engineering & Institute of Biotechnology and Chemical Engineering, I-Shou University, Kaohsiung, Taiwan; 8 Department of Electronic Engineering, National Kaohsiung University of Applied Sciences, Kaohsiung, Taiwan; 9 Institute of Clinical Medicine, Kaohsiung Medical University, Kaohsiung, Taiwan; 10 Kaohsiung Municipal Ta-Tung Hospital, Kaohsiung, Taiwan

**Keywords:** Single nucleotide polymorphism, Genetic algorithm, SNP interaction, Breast cancer

## Abstract

**Background:**

*ORAI1* channels play an important role for breast cancer progression and metastasis. Previous studies indicated the strong correlation between breast cancer and individual single nucleotide polymorphisms (SNPs) of *ORAI1* gene. However, the possible SNP-SNP interaction of *ORAI1* gene was not investigated.

**Results:**

To develop the complex analyses of SNP-SNP interaction, we propose a genetic algorithm (GA) to detect the model of breast cancer association between five SNPs (rs12320939, rs12313273, rs7135617, rs6486795 and rs712853) of *ORAI1* gene. For individual SNPs, the differences between case and control groups in five SNPs of *ORAI1* gene were not significant. In contrast, GA-generated SNP models show that 2-SNP (rs12320939-GT/rs6486795-CT), 3-SNP (rs12320939-GT/rs12313273-TT/rs6486795-TC), 5-SNP (rs12320939-GG/rs12313273-TC/rs7135617-TT/rs6486795-TT/rs712853-TT) have higher risks for breast cancer in terms of odds ratio analysis (1.357, 1.689, and 13.148, respectively).

**Conclusion:**

Taken together, the cumulative effects of SNPs of *ORAI1* gene in breast cancer association study were well demonstrated in terms of GA-generated SNP models.

## Background

Single nucleotide polymorphisms (SNPs) are one of the most common variants in human genome [1]. Currently, SNPs have been applied to the association studies for complex diseases [2-4]. Genome-wide association studies (GWAS) can identify the several SNPs predisposing to many diseases [5-8]. Although GWAS covers human genome-wide SNPs, many SNPs of non-significance are commonly ignored. Recently, the possible jointed effects of gene-gene interactions are gradually uncovered in predicting many disease risks [9-12]. However, when simultaneously evaluate the complex interactions amongst huge SNPs, these interactions are complex and it may need the help of new strategy [13] or computation [14].

Similarly, the non-GWAS association studies have the similar condition to ignore the possible gene-gene interactions. For example, several individual SNPs of the ORAI calcium release-activated calcium modulator 1 (*ORAI1*) gene have reported to be involved in breast cancer susceptibility [15]. However, the possible SNP-SNP interactions of *ORAI1* gene associated with breast cancer were not addressed. Different computational analyses have been introduced to examine SNP-SNP interaction in many association studies [14,16-23]. Genetic algorithm (GA) is potential for feature selection for genome-wide scale datasets [24] and may apply to compute the difference between case and control groups to identify good models from the huge SNP combinations as well as tagSNP selection [25].

To address the possible SNP-SNP interaction in breast cancer susceptibility, five tagSNPs (rs12313273, rs6486795, rs7135617, rs12320939, and rs712853) of *ORAI1* gene were selected in this study. Therefore, we introduced the GA to optimizing the analyses of SNP-SNP interactions of *ORAI1* gene associated with breast cancer. GA is used to identify the best SNP models (SNP combinations with genotypes) with maximum frequency difference between breast cancer and control groups. Therefore, the best GA-generated SNP models of *ORAI1* gene may be useful for predicting the breast cancer risk.

## Methods

### Data set collection

The case and control subjects are 345 female breast cancer patients and 290 female normal controls where the recruitment was approved by Cancer Center of Kaohsiung Medical University Hospital. The genotype dataset of breast cancer patients of five tagSNPs (rs12313273, rs6486795, rs7135617, rs12320939, and rs712853) of *ORAI1* gene with minimum allele frequency (MAF) >10% obtained from our previous study [15]. For normal controls, samples of were collected in current study and SNP genotyping was performed as described [15].

### Genetic algorithm

The GA [26] is a well-known evolutionary algorithm, and it has been applied for solving the complex problems in several fields. GA simulates the natural evolution to generate solutions of complex problems, including selection, crossover, mutation, and inheritance. The process of GA has six steps: (1) initializing population, (2) evaluate chromosome values, (3) select two parents using selection operation, (4) crossover operation, (5) mutation operation, and (6) replacement operation.

A population in first step is initialized according encoding schemes of problem. Second step aims to evaluate value of chromosomes in population using fitness function. Third step use the evaluated value of chromosomes to select the two good parents for generating two offspring (step 4). Then firth step is probabilistic to mutate two offspring. Final step is used to improve the value of population. Thus repeat of steps 2 to 6 in several generations can effectively search the good values of chromosomes in population, and a best chromosome in population is regarded to best solution. Algorithm 1 shows the GA pseudo-code, and the below section is detailed to explain the processes of six steps.

Algorithm 1: Genetic algorithm pseudo-code.

### Encoding schemes

A population consists of the several possible solution of problem. The possible solution in GA is named a chromosome that is a set *C* = {*c*_1_, …, *c*_*d*_}. In this study, a chromosome indicates a possible model of associations between SNPs. All combinations of SNPs and genotypes can be represented a set *A* = *S* × *G* = {(*s*, *g*)| *s*∈*S* and *g*∈*G*} where *S* is a set of SNPs and *G* is a set of genotypes. For example, we assume an *S* contains two SNPs and a *G* contains three genotypes, i.e., *S* = {s_1_, s_2_} and *G* = {g_1_, g_2_, g_3_}. All possible subsets can be represented *A* = *S* × *G* = {(s_1_, g_1_), (s_1_, g_2_), (s_1_, g_3_), (s_2_, g_1_), (s_2_, g_2_), (s_2_, g_3_)}. Each subset in *A* represents the selected SNP and their genotype. A chromosome is defined *C* = {*c*_1_, …, *c*_*d*_ | *c*_*i*_, *c*_*j*_ ∈*A*, *c*_*i*_ ≠ *c*_*j*_, 1 ≤ *i ≠ j* ≤ *d*} where *d* is the association model size. A possible chromosome in above example can be assigned as *C* = {(s_1_, g_1_), (s_2_, g_2_)}; it means a model that includes the genotype “AA” of first SNP and the genotype “Aa” of second SNP.

### Fitness function

A value of chromosome *C* can be evaluated by computing the fitness function; it facilitates GA for eliminating the worst chromosomes of population in each generation. In this study, a total number difference between case data and control data at a model is used to design a fitness function. Equation 1 is used to check a model whether a SNP is repeatedly selected or not. If a SNP is repeatedly selected in a *C*, the value of *C* is evaluated to zero. If it is not, Equation 2 is used to calculate the total number difference between cases and controls at a model. In Equation 2, the max_P and max_N are a total number of case data and a total number of control data, respectively. The *P* and *N* are respectively represented the set of case data and a set of control data; *P*_*i*_ is the *i*^th^ patient sample in case data and *N*_*i*_ is the *i*^th^ normal sample in control data. Equation 3 is used to evaluate whether all factors in a model are included in a set of sample. If a sample includes the model, the Equation 3 returns one value into Equation 2; whereas, it returns zero value.

(1) $$f\left(C\right)=\left\{\begin{array}{cc} 0\; & \mathrm{if}\underset{s_{i}\in C}{\cap }s_{i}\neq \varphi \\ d\left(C\right) & \mathrm{if}\underset{s_{i}\in C}{\cap }s_{i}=\varphi \end{array}\right)$$

(2) $$d\left(C\right)=\frac{\sum_{i=1}^{\max\_P}m\left(C,P_{i}\right)}{\max\_P}-\frac{\sum_{i=1}^{\max\_N}m\left(C,N_{i}\right)}{\max\_N}$$

(3) $$m\left(X,Y\right)=\left\{\begin{array}{cc} 0 & \mathrm{if}\;X⊄Y\\ 1 & \mathrm{if}\;X\subseteq Y \end{array}\right)$$

### Selection operation

Selection operation aims to select the good chromosomes for generating the great offspring; the selected chromosomes name parents. Selection operation in this study uses a rank-based tournament scheme for selecting the two parents. The operation uses fitness function to evaluate all chromosomes of a population *P* = {*C*_1_, …, *C*_*i*_|*i* is population size}, and all values in *P* are recorded into a set *R* = {*r*_1_, …, *r*_*i*_| *i* is population size}. These values represent chromosome ranks. Then *R* is sorted from the big value to small value, i.e., *r*_1_ ≥ *r*_2_ ≥ *r*_*i*_. Thus the *r*_1_ and *r*_2_ with corresponding *C*s in *P* are two selected parents.

### Crossover operation

Crossover operation is used to generate the offspring from the parents, and the operation use a uniform crossover scheme. Uniform crossover firstly generate a binary mask set *B* = {*b*_1_, …, *b*_*i*_| *b*∈[0,1], *i* = |*C*|}; a binary value at *b* is randomly generated. The one value of *b*_*j*_ indicates that *j*^th^ elements of two parents are must be exchanged; the zero value represents the no exchange. Two offspring are generated by exchanging the elements of two parents according the binary mask set, and the offspring are represented *C’*. For example, let a generated binary mask *B* = {1, 0, 1, 0} and two parents *C*_1_ = {(s_1_, g_1_), (s_2_, g_2_), (s_5_, g_1_), (s_3_, g_3_)} and *C*_2_ = {(s_1_, g_3_), (s_2_, g_1_), (s_4_, g_2_), (s_3_, g_2_)}. The generated two offspring are *C’*_1_ = {(s_1_, g_3_), (s_2_, g_2_), (s_4_, g_2_), (s_3_, g_3_)} and *C’*_2_ = {(s_1_, g_1_), (s_2_, g_1_), (s_5_, g_1_), (s_3_, g_2_)}, respectively.

### Mutation operation

Mutation operation can facilitate the diversity of chromosomes in population, and avoid population for trapping the local optimal. The operation uses a one-point mutation operation. A mutation point set *M* = {*m*_1_, …, *m*_*i*_| *m*∈[0,1], *i* = |*C*|} is used to indicate the mutation points in the offspring *C’*. Each binary value in *M* is randomly generated according to the mutation probability. The one value of *m*_*j*_ represents that *j*^th^ element of *C* do the mutation; the zero value represents the no mutation. The mutation randomly generates a possible *c* element, where *c*∈A, to replace the original element at a mutation point. For example, let a generated mutation point *M* = {0, 0, 1, 0} and offspring *C’*_1_ = {(s_1_, g_3_), (s_2_, g_2_), (s_4_, g_2_), (s_3_, g_3_)}. Suppose the number of SNPs is five, a possible set is *E* = {(s_4_, g_1_), (s_4_, g_2_), (s_4_, g_3_), (s_5_, g_1_), (s_5_, g_2_), (s_5_, g_3_)}. After mutation the offspring could be *C’*_1_ = {(s_1_, g_3_), (s_2_, g_2_), (s_5_, g_3_), (s_3_, g_3_)}.

### Replacement operation

Replacement operation aims to gradually improve value of population. The generated two offspring *C’*_1_ and *C’*_2_ are evaluated by fitness function, and are used to compare the value to all chromosomes. When an offspring is higher value than a chromosome of population, it replaces the chromosome; otherwise, the offspring is deleted.

### Parameter settings

In the GA parameters, both of the exchange probabilities in the tournament selection and uniform crossover are 1.0. The exchange probability of a one-point mutation is 0.1. The population size is 50, and the number of generations is 100.

### Statistical analysis

All statistical value is computed using SPSS version 19.0 (SPSS Inc., Chicago, IL). Odds ratio (*OR*) with 95% confidence interval (CI) is used for measuring a single SNP and the model of association between SNPs; a *P* value of < 0.05 is considered statistically significant difference between the cases and controls.

## Results

### Data collection

The complete genotype data set is available at http://bioinfo.kmu.edu.tw/BRCA-ORAI1-5SNPs.xlsx. Based on these data, the GA-generated SNP models to address the possible SNP-SNP interaction in *ORAI1* gene were evaluated in terms of breast cancer association later.

### Comparison of patients and normal in terms of effect of single SNP

Table 1 shows the occurrence of breast cancer for five SNPs in *ORAI1* gene. The genotype with major allele (G in rs12320939; T in rs12313273; G in rs7135617; T in rs6486795; and T in rs712853) is regarded as the reference for analyzing breast cancer risks in terms of single SNPs. Minor allele is selected according the dbSNP database of NCBI (National Center for Biotechnology Information). No significant differences between the breast cancer patients and controls in all genotypes for each single SNP were found.

**Table 1:** The performance of five individual SNPs for breast cancer and control groups

**SNP ID**	**Genotype**	**Case (%)**		**Control (%)**		***p*-value**
1. rs12320939	GG	97	(28.12)	79	(27.24)	
	GT	181	(52.46)	140	(48.28)	0.785
	TT	67	(19.42)	71	(24.48)	0.248
2. rs12313273	TT	183	(53.04)	161	(55.52)	
	TC	142	(41.16)	100	(34.48)	0.189
	CC	20	(5.80)	29	(10.00)	0.107
3. rs7135617	GG	103	(29.86)	94	(32.41)	
	GT	187	(54.20)	145	(50.00)	0.367
	TT	55	(15.94)	51	(17.59)	0.947
4. rs6486795	TT	137	(39.71)	121	(41.72)	
	TC	173	(50.14)	126	(43.45)	0.260
	CC	35	(10.14)	43	(14.83)	0.204
5. rs712853	TT	154	(44.64)	128	(44.14)	
	TC	158	(45.80)	134	(46.21)	0.904
	CC	33	(9.57)	28	(9.66)	0.942

### Identification of the best model of SNPs association with maximum frequency difference between breast cancer and control groups

During GA processing, the best ten models of two SNP combinations with genotypes (2-SNP models) were demonstrated in Table 2. In these 2-SNP models, the SNPs (1, 4) with genotype 2-2, i.e., [rs12320939-GT]-[rs6486795-TC], possessed the maximum frequency difference (7.20%) between the breast cancer and control groups, namely the best 2-SNP model. Similarly, the best GA-generated SNP models involving three to five SNP were shown in left side of Table 3.

**Table 2:** The best 10 models in 2-SNP order association

**Combined SNP number (specific SNPs)**	**SNP Genotypes**	**Case no.**	**Control no.**	Difference (%)*
SNP(1,4)	2-2	145	101	7.20
SNP(2,4)	2-3	22	1	6.03
SNP(3,4)	2-2	121	85	5.76
SNP(2,5)	2-3	83	54	5.44
SNP(2,4)	3-2	65	40	5.05
SNP(1,5)	2-3	81	55	4.51
SNP(4,5)	2-3	81	55	4.51
SNP(3,5)	2-3	78	53	4.33
SNP(2,3)	3-3	38	23	3.08
SNP(4,5)	2-2	77	56	3.01
SNP(1,3)	2-2	147	116	2.61

*Difference (%) = Frequency of cases (breast cancer group) - Frequency of controls.

**Table 3:** The odds ratio of the best SNP models associated with breast cancer

**Combined SNP no. (specific SNPs)**	**SNP Genotypes**	**Case no./control no.**	**Cancer (%)**	Difference (%)*	**Odds ratio (95%CI)**	***P *** **value**
2-SNP (1-4)	2-2	145/101	58.94	7.20	1.357	0.064
	Others	200/189	51.41		(0.983-1.873)	
3-SNP (1-2-4)	2-1-2	58/31	65.17	6.12	1.689	**0.028**
	Others	287/259	52.56		(1.058-2.694)	
4-SNP (1-3-4-5)	2-2-2-1	78/53	59.54	4.33	1.306	0.180
	others	267/237	52.98		(0.884-1.930)	
5-SNP (1-2-3-4-5)	1-2-3-1-1	14/0	100.00	4.06	13.148	**0.013**
	Others	331/290	53.30		(1.726-100.142)	

Bold letters: The SNP models associated with breast cancer are significant (*P* value < 0.05).

*Difference (%) = Frequency of cases (breast cancer group) - Frequency of controls.

### Odds ratio analysis to identify the best models of SNP associations associated with high breast cancer

Table 3 shows five best models of association involving two to five SNPs. Odds ratio analysis shows the performance (*OR*, 95% CI, and *P* value) of five types of the best models (2- to 5-SNP models) addressing the breast cancer association. The 2-SNP model, i.e., SNPs (1, 4) in genotype 2-2, indicates the borderline significance with breast cancer (*OR*: 1.357, *P* = 0.064). The 3-SNP model, i.e., SNPs (1, 2, 4) in genotype 2-1-2, indicates three SNPs (rs12320939, rs12313273, and rs6486795) have a significant association when their genotypes are GT, TT, and TC, respectively (*OR*: 1.689, *P* = 0.028). The 5-SNP model, SNPs (1, 2, 3, 4, 5) in genotype 1-2-3-1-1, indicates all SNPs (rs12320939, rs12313273, rs7135617, rs6486795, and rs712853) have a strongly association when their genotypes are GG, TC, TT, TT, and TT, respectively (*OR*: 13.148, *P* = 0.013).

## Discussion

GA is a robust non-parametric method that detects nonlinear interactions amongst multiple discrete genetic factors. The advantage of GA is that the method can directly search the good models from the huge number of possible combinations without the training data set. In this study, the fitness function is designed based on the unbalanced data set to compute the difference between case data set and control data set. The function can effectively measure high-risk to search the good model in real data set.

In current study, the *OR* values of 2- to 3-SNP models are larger than 1 but small, suggesting that the cumulative effect of these four SNPs (rs12320939, rs7135617, rs6486795, and rs712853) are weak. When five SNPs included, the *OR* value is 13.148, indicating that the cumulative effect of 5-SNP model becomes strong. This unstable cumulative effect of SNP combinations in SNP models may be partly explained by the experiment design that these five SNPs were only derived from a single gene *ORAI1*. Because breast cancer is a kind of multigene disease [27-30], therefore, SNPs derived from more genes included in association studies may reveal the cumulative effect effectively [9,11,12,31-33]. Accordingly, the differential performance of the cumulative effects of SNPs from single gene and multigene is worth of further investigation in future.

The computational complexity of GA is calculated by a fitness function of computation. Suppose *n* iterations is implemented in a test, the computational complexity of GA is O(*n*) which represents the big-O in complexity analysis. GA in search of good association model has the below advantages: (1) GA effectively identify the high-risk models in high-order interaction, (2) the best model with statistical significant can be fast identified, and (3) it only has two parameters to need setting and is easily to fulfil for searching the good model. Further, GA is able to analyze high order SNP interactions amongst the huge number of SNPs from GWAS and pharmacogenomics studies in our experiences.

## Conclusions

Although the polymorphisms of *ORAI1* gene have been reported to associate with inflammatory diseases [34-36], effects of SNP-SNP interaction to diseases are still unclear. In this study, the GA successfully identified appropriate models of SNP-SNP interactions in breast cancer association study in terms of five SNPs in *ORAI1* gene. The resulting SNP models can predict the breast cancer susceptibility more effective than the individual SNPs. This methodology can also apply to any kinds of SNP association studies, such as GWAS, pharmacogenomics and others. Therefore, the possible cumulative effect of SNP combination will be uncovered by this methodology*.*

## Competing interests

The authors have no competing interests to declare.

## Authors’ contributions

W-CC and M-FH carried out the molecular genetic studies. W-CC and Y-YF drafted the manuscript. C-HY instructed Y-DL to perform SNP interaction analysis by algorithm. Y-YF, H-WC, and L-YC performed statistics analyses. H-WC, C-HY, and M-FH were involved in discussion and editing the manuscript. All authors read and approved the final manuscript.
